# Chemical Modification of Glycosaminoglycan Polysaccharides

**DOI:** 10.3390/molecules26175211

**Published:** 2021-08-27

**Authors:** Lais C. G. F. Palhares, James A. London, Aleksandra M. Kozlowski, Emiliano Esposito, Suely F. Chavante, Minghong Ni, Edwin A. Yates

**Affiliations:** 1 Programa de Pós-graduação em Bioquímica e Biologia Molecular, Departamento de Bioquímica, Universidade do Rio Grande do Norte (UFRN), Natal 59012-570, Brazil; laiscgf.palhares@gmail.com (L.C.G.F.P.); chavantesu@hotmail.com (S.F.C.); 2 Department of Biochemistry and Systems Biology, ISMIB, University of Liverpool, Crown Street, Liverpool L69 7ZB, UK; jlondon@liverpool.ac.uk; 3 Department of Chemistry and Chemical Engineering, Forest Products and Chemical Engineering, Chalmers University of Technology, Kemigården 4, 412 58 Göteborg, Sweden; aleksandra.kozlowski@chalmers.se; 4 Instituto di Richerche Chemiche e Biochemiche, ‘G.Ronzoni’, Via G Colombo 81, 20133 Milano, Italy; esposito@ronzoni.it (E.E.); niminghong@ronzoni.it (M.N.)

**Keywords:** glycosaminoglycans, polysaccharides, sulfation, chemical modification

## Abstract

The linear anionic class of polysaccharides, glycosaminoglycans (GAGs), are critical throughout the animal kingdom for developmental processes and the maintenance of healthy tissues. They are also of interest as a means of influencing biochemical processes. One member of the GAG family, heparin, is exploited globally as a major anticoagulant pharmaceutical and there is a growing interest in the potential of other GAGs for diverse applications ranging from skin care to the treatment of neurodegenerative conditions, and from the treatment and prevention of microbial infection to biotechnology. To realize the potential of GAGs, however, it is necessary to develop effective tools that are able to exploit the chemical manipulations to which GAGs are susceptible. Here, the current knowledge concerning the chemical modification of GAGs, one of the principal approaches for the study of the structure-function relationships in these molecules, is reviewed. Some additional methods that were applied successfully to the analysis and/or processing of other carbohydrates, but which could be suitable in GAG chemistry, are also discussed.

## 1. Introduction

Among polysaccharides, the glycosaminoglycans (GAGs) are notable because of their participation in diverse biological events and their many biomedical applications. Their structural analysis can also depend on their chemical derivatization. The availability of methods for the chemical modification of GAGs enables the preparation of products with improved therapeutic potential [1]. Several studies describing or utilizing the modification of GAGs for structural analysis or biotechnological purposes have been reported [2,3,4,5,6,7]. In this review, we address chemical methods for the modification of GAG polysaccharides, concentrating largely on modifications within the polysaccharide chain that result in naturally-occurring residues being produced, albeit in potentially unnatural sequences, rather than modifying the structure through the addition of new chemical groups. The modifications at reducing ends or the non-reducing terminus, e.g., the formation of a double bond by, for example, lyase enzyme action, with a few exceptions, have largely been excluded. We also discuss selected methods taken from the wider chemical literature that, in future, could be applied to modify GAGs. 

### 1.1. Structure and Classes of Glycosaminoglycans

GAGs represent a complex group of macromolecules found in the extracellular matrices and tissues of vertebrates and invertebrates [8], and can be localized inside and on the surface of cells. These linear anionic polysaccharides are comprised of repeating disaccharide units, consisting of a hexosamine (D-glucosamine or D-galactosamine) residue bound to a non-nitrogenated sugar residue (D-glucoronate, L-iduronate, or D-galactose) [9]. The identities of these residues, the number and position of the sulfate groups, as well as the positions and configurations of glycosidic bonds, [10] distinguish the different classes of GAGs, i.e., chondroitin-4 and -6 sulfate (CS, also known as chondroitin sulfate-A and -C), dermatan sulfate (DS), heparan sulfate (HS), heparin (Hp), hyaluronic acid (HA), and keratan sulfate (KS) [11]. 

### 1.2. General Properties 

**Figure 1:**
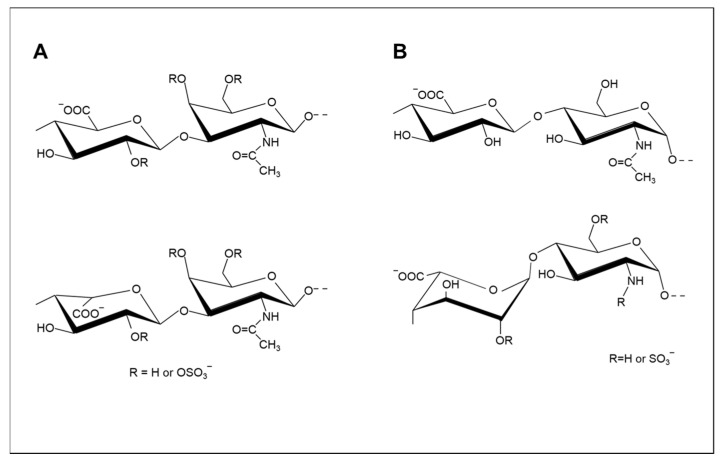
Typical repeating disaccharides of chondroitin ((**A**), upper) and dermatan ((**A**), lower) sulfates ((**A**), lower) and repeating disaccharides of heparan sulfate ((**B**), upper) and heparin ((**B**), lower).

From a structural point of view, GAGs are linear anionic polysaccharides composed of multiple repeating disaccharides (Figure 1), with numerous sequence variants giving rise to heterogeneous populations. For example, octasaccharides from HS could have over 1,000,000 hypothetical sulfation arrangements [12] and, if only a small proportion of these possibilities are observed, this still represents considerable heterogeneity. This wide range of structural variations is accompanied by extensive molecular weight variation, different electronegativity characteristics, and sulfate clustering to generate a distinct binding potential and distinct regions within the chains. While the molecular weight of purified Hp is typically around 12–16 kDa, HA can reach 10,000 kDa [13], but, within a particular GAG population, varying molecular weights are usually observed [14]. 

The biological activities of GAGs arise predominantly from their interaction with proteins. The binding forces involved are usually dominated by ionic interactions, especially between the basic amino acids, lysine and arginine on the protein surface. These interactions are supported by hydrogen bonding involving polar residues, which are crucial, together with a degree of shape complementarity, to the formation of GAG-protein complexes between, for example, growth factors and their receptors [15]. High molecular weight HA can stimulate cell surface CD44 clustering, while small oligosaccharides attenuate the clustering effect [16]. Besides the variation in molecular weight, the polyelectrolyte properties provide a unique fingerprint. In Hp, for example (particularly in the active pharmaceutical product), the *N*-sulfated residues significantly outweigh the *N*-acetylated regions, resulting in an average sulfation degree of ~2.7, equating to almost 40 sulfate groups per polysaccharide chain of 10 kDa. The overall negative charge density of the polysaccharides is further increased by carboxyl groups, making them the most negatively charged biomacromolecules. Furthermore, owing to the low pK_a_ values (≤3.2 for carboxylates and substantially lower for sulfate groups), these anionic groups are completely deprotonated at physiological pH. Their high charge density and conformational flexibility allow GAGs to display diverse protein binding activity, which, to a significant extent, is driven by these charge interactions. 

### 1.3. Biosynthesis

Glycosaminoglycan sequences in vivo are regulated in a tissue- and organism-specific manner that can be modulated according to physiological and pathological conditions [17,18,19]. The mechanism by which GAG biosynthesis is regulated, however, remains an open question and is the subject of intense research. Distinct from proteins and nucleic acids, the biosynthesis of GAGs is not template driven and the strict, sequential application of biosynthetic enzymes cannot account for all of the structures observed [20,21]. Furthermore, a degree of post-biosynthetic modification is possible, with the sulf enzymes being responsible for the removal of 6-*O*-sulfate groups from glucosamine residues. GAG-binding proteins of functional significance include cytokines and chemokines, enzymes and enzyme inhibitors, extracellular matrix proteins, and membrane receptors.

The biosynthesis of GAG chains is initiated by a common GAG-protein linkage tetrasaccharide sequence, with D-GlcA β (1-3) D-Gal β (1-3) D-Gal β (1-4) D-Xyl β(1-*O*-(Ser) attached at the serine residues of their respective core proteins. The GAG-attachment sites have the consensus peptide sequence Ser-Gly/Ala-X-Gly, where X represents any amino acid [22]. The linkage region tetrasaccharide is formed by the stepwise addition of each sugar unit to the serine residue by glycosyltransferases that follows the order: xylosyltransferase (XylT), galactosyltransferase I (GalT-I), galactosyltransferase II (GalT-II), and glucuronyltransferase I (GlcAT-I). The subsequent addition of the appropriate disaccharide units affords the different types of GAGs. The enzymes employed during their biosynthesis include the sulfotransferases and epimerases, with the latter being responsible for the introduction of the L-iduronate (L-IdoA) residues in HS, Hp, and DS [23]. The synthesis of HS/Hp onto this linkage region is triggered by addition of the fifth sugar, α-D-GlcNAc, by the enzyme α-*N*-acetyl glucosaminyltransferase I (GlcNAcT-I). In contrast, the transfer of a β-D-GalNAc residue to the linkage region initiates and determines the synthesis of CS/DS chains by the corresponding enzyme β-*N*-acetyl galactosaminyltransferase I (GalNAcT-I) [24]. The relationship between GAG structure and some of the many biological activities with which they are involved was reviewed by [25]. 

The presence of specific GAG biosynthetic enzymes in organisms is related to the emergence of multicellularity and tissue organization, with GAGs being a characteristic of the metazoan lineage. Moreover, a correlation between the complexity of an organism and the number of HS sulfotransferase isoforms is evident [26], suggesting the importance of different degrees and patterns of sulfation to biological complexity. 

### 1.4. Glycosaminoglycan Interactions with Proteins 

The interaction of GAGs with a large number of proteins were reported, especially for HS/Hp [27]. These interactions proceed largely through a combination of charge interactions between the basic amino acid residues of the protein, i.e., arginine, lysine and, under appropriate conditions, histidine, with the negative sulfate and carboxyl groups of GAGs. One important family of signaling molecules, fibroblast growth factors (FGFs), has been mapped in some detail [28,29,30]. The significant number of hydrogen bonds and weaker interactions also play a role, while the overall conformation and flexibility of the GAGs provide complementary surfaces with appropriately arranged pendant groups, although their topologies are much more difficult to assess in the absence of detailed structural studies, which are hindered by the complexity of GAGs and a number of technical challenges [31]. Thus, while some progress towards understanding the dependence of specificity on GAG structure has been made (reviewed; [21]), the potential relationship to HS biosynthesis and regulation, although long appreciated [32] remains to be fully explored. Interestingly, it was suggested recently that one role of GAGs may be to provide a clearance mechanism for misfolded protein [33]. It is not our intention, in the present review, to survey the large number of biological activities with which GAGs are associated, but we will refer to relevant examples throughout the text.

The hypothesis that the diverse biological activities of GAGs can be modulated, or even have entirely new activities induced, was borne out of numerous studies. These have usually involved extracted, chemically, or enzymatically modified GAGs, as well as other types of structurally-similar compounds with therapeutic potential [2,5,6,7,34,35]. The particular chemistry of this class of polysaccharides, subject to restrictions imposed by their highly functionalized nature and consequently limited solubility in conventional solvents, has developed relatively slowly. Nevertheless, there now exists a battery of established methods for their controlled manipulation, which we attempt to summarize here. Several methods for additional modifications, taken from other classes of polysaccharides and the wider chemical literature, which the authors considered to be potentially applicable to GAGs, are also included. In this review, the International Union of Pure and Applied Chemistry recommended spelling of sulfate was adopted throughout.

## 2. Chemical Modifications 

Glycosaminoglycans are highly functionalized, bearing combinations of hydroxyl, *O*- and *N*-sulfate, *N*-acetyl, carboxylates and, occasionally, free amine groups. Carboxylate and sulfate groups lend the polysaccharides their anionic, water soluble character, but also limit their solubility in most conventional solvents. Nevertheless, it is possible, to form organic salts by neutralizing the acidic forms of polysaccharides (generated by treatment with acidic cation exchange resin) with the hydroxides of tertiary or quaternary amines, followed by freeze-drying to access alternative solvent systems. This was exploited widely to carry out reactions in dimethyl sulfoxide (DMSO) and dimethyl formamide (DMF), but other solvents can undoubtedly be explored using this approach. One example is ionic liquids [36], which have enabled development of alternative protocols for the mass spectrometry of GAGs [37,38,39] and warrant wider investigation. Supercritical carbon dioxide was also employed as a means of extracting extracellular material, including GAGs [40]. Methods for the removal of ionic liquids have been described for non-GAG carbohydrates, consisting of anhydrous ethanol washes and subsequent filtration, centrifugation (provided that aqueous buffers are also involved), and the use of silica columns [41,42,43]. The modification of non-GAG carbohydrates in ionic liquids employs a variety of cation and anion components, with a wide range of combinations available for the potential modification of GAGs. Some have also been used previously to modify other charged linear polysaccharides [44,45]. These novel solvents can also be recycled, allowing for ‘greener’ chemical modification protocols, although the potential toxicity of ionic liquids need to be considered [46,47]. An additional novel replacement for dipolar aprotic solvents, such as DMF, dihydrolevoglucosenone (Cyrene), has been developed and recently tested, although not yet with a carbohydrate starting material [48,49,50]. These areas are in their relative infancy, but nevertheless hold promise and will be worth exploring in more detail. Their distinct properties are likely to influence the reactivity of the GAGs through altered hydrogen-bonding (both intramolecular and with the solvent), and the resulting changes in conformation may be the cause of any differences in selectivity that are observed.

### 2.1. Modification of Sulfation Patterns 

Sulfated polysaccharides play important roles in numerous physiological and patho-physiological processes, including the coagulation cascade, viral transmission, and as antioxidants [51]. Introducing or removing sulfate groups in polysaccharides leads to different negative charge densities and resultant structure-property-activity relationships. Thus, it is of interest both for basic study and for the development of new products. In interpreting results from investigations of biological activities employing modified GAGs, it should be borne in mind that conformational changes usually accompany the removal of sulfate groups [21]. The sulfate group, an analogue of sulfuric acid, carries an invariant negative charge across physiological pH ranges, enabling it to bind electrostatically to positively charged biomolecules. It can interact with water molecules to increase and maintain tissue hydration [52]. Some examples of the use of modified GAGs to probe biological activities include studies of fibroblast growth factors [28], Alzheimer’s β-secretase [53], and the inhibition of viral invasion [54]. 

Regioselective sulfation and desulfation of naturally occurring polysaccharides and their synthetic analogues is challenging [55]. Nevertheless, sulfation and desulfation processes are both widely used to modify GAG structures and can be followed by subsequent modifications, with the underlying aim usually being to alter, in some way, the biological activity of the GAG. 

#### 2.1.1. Selective Addition of Sulfate Groups

The extent of GAG sulfation and, especially the sulfation pattern, plays an essential role in determining their ability to bind to a wide range of biologically relevant proteins, including growth factors, as was shown for sulfated HA and oversulfated CS towards transforming growth factor-β1 (TGF-β1) [56] and for oversulfated CS with neuronal growth factors, respectively [17]. In the case of *O*-sulfation, the process involves derivatization of the primary and/or secondary hydroxyl groups (with some level of control), an example of which was achieved during sulfation of the capsular *E. coli* K5 polysaccharide [57]. Higher selectivity can be achieved for modifications at the primary hydroxyl groups of glucosamines, while, in the case of N-sulfation, substitution of free amines is possible with essentially complete selectivity [58]. *N*-sulfated, *O*-desulfated heparin (tributylammonium salt) was regioselectively 6-*O*-sulfated by treating it with pyridine sulfur trioxide complex (Py.SO_3_) in DMF at −10 °C for 1 h [59]. *N*-sulfated heparosan was also *O*-sulfated in DMF, employing a sulfur trioxide complex with pyridine under similar conditions, which provided sulfation at position 6 of *N*-sulfated GlcNAc units together with some sulfation of GlcA residues either at the *O*-2 or *O*-3 positions [60]. Sulfation through treatment with sulfuric and chlorosulfonic acids (CSA) is another strategy, which is commonly applied to increase the sulfate/carboxylate molar ratio; however, beyond overall sulfation, this approach offers little regioselectivity and can cause depolymerization. To overcome this drawback, Wang et al. developed a *dimethylaminopyridine/N, N′ dicyclohexylcabodiimide* (DMAP/DCC) catalyzed CSA sulfation method for polysaccharides [61]. 

Many studies have confirmed that the biological activities of polysaccharides can be improved by GAG modification. For example, N-sulfated DS and CS showed inhibitory effects on the TGF-β1 gene [62], whereas over-sulfated heparins presented somewhat reduced anticoagulant activity, while maintaining antithrombotic and lipase potential [63]. With increased sulfation, however, comes the risk of introducing other biological activities or enhancing existing (possibly undesirable) activities, and even inducing the emergence of new, unwanted biological effects. A stark reminder of the dangers associated with the introduction of off-target effects is the contamination of pharmaceutical Hp supplies with over-sulfated CS that, in 2007 and 2008, led to numerous deaths [64,65].

Polysaccharide sulfation in an aqueous solution under conditions sufficiently mild to avoid rapid depolymerization, although conceivable, is likely to proceed slowly and require longer times to achieve equilibrium [66]. A potential alternative may, again, be ionic liquids, many of which are miscible with both water and organic solvents. These also offer good solubility for polysaccharides and provide a homogenous reaction system. A significant increase in both molecular weight and sulfur content was observed for a GAG-like compound of marine origin, achieved with a one-step chemical sulfation process using ionic liquid, i.e., 1-butyl-3-methylimidazolium chloride (BMImCl), as a reaction medium, without undesired degradation of the polysaccharide backbone [67]. In this case, chemical sulfation provided a pro-chondrogenic effect and, when added to a chondrogenic differentiation medium, it enhanced the chondrogenic differentiation of mesenchymal stem cells, which is considered an attractive source of cells for cartilage engineering [68]. Moreover, in this process, the sulfation level could be modulated by varying the temperature or the reaction time. 

Some methods, taken from classical protecting group approaches for the synthesis of small carbohydrates, were also applied to improve regioselective sulfation. For instance, a 4,6 benzylidene acetal protection of glucosamine residues, followed by sulfation with sulfur trioxide-pyridine complex in DMF and de-protection, afforded the development of chondroitin sulfate-E (CS-E)-like compounds [69]. The combination of multi-step processes can be applied to CS-A and CS-C to obtain different sulfation patterns. Benzylidene ring installation and acetylation in a one-pot fashion, followed by oxidative benzylidene cleavage, sulfation, and global de-protection, were also used to achieve 3-*O*-sulfation of GlcA groups in CS compounds [70]. An additional possibility, as yet to be applied to GAGs, is the pivaloyl protecting group. This group was used to enable regioselective sulfation, employing pyr·SO_3_ in DMF, of another linear sulfated carbohydrate family, carrageenans, to generate a panel of variously modified samples with applications analogous to those of the GAGs. The subsequent deprotection and activated partial thromboplastin time (aPTT) coagulation activity measurements of the variably sulfated carrageenan panel indicated widely varying coagulation times similar, in some cases, to that of Hp [71]. 

When using DMF, microwave assistance during *O*-sulfation of a protected Hp disaccharide reduced the processing times, from days to 1 h, and produced high reaction yields. Rapid reaction times were also reported for protected Hp tetra- and hexa-saccharide, with yields greater than 80% within 20 min [72]. Similar reaction times and high yields were reported when using microwave-assisted pyridine and triethylamine solvent systems for simultaneous *O*- and *N*-sulfation of Hp and heparan sulfate-like oligosaccharides with trimethylamine sulfur trioxide as the sulfating agent [73].

In addition to direct GAG sulfation, analogous (but uncharged) groups can also be added. An example from outside of the GAG field is cellulose derivatization. Sulphoethylation generates samples with increased viscosity, tensile strength, and pH stability compared to pre-existing, carboxymethyl derivates. Sulphoethylation in 2-propanol solvated sodium vinylsulphonate, sodium chloroethanesulphonate monohydrate, or sodium bromoethanesulphonate monohydrate, generated variable yields, with sodium vinylsulphonate proving the most effective [74,75]. An interesting recent development is the application of tributylsulfoammonium betaine (TBSAB) to achieve *N*- and *O*-sulfations of smaller molecules, amino acids, and peptides [76,77]. It will be interesting to discover whether these can be applied to GAGs.

#### 2.1.2. Selective Removal of Sulfate Groups

**Figure 2:**
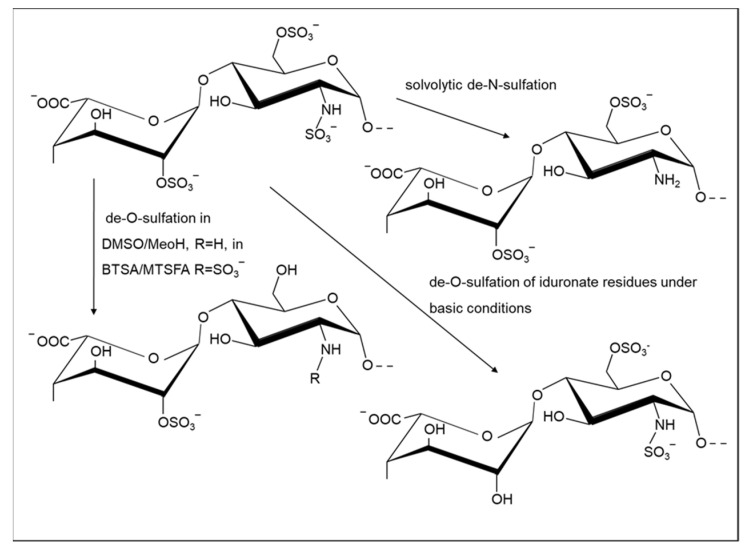
De-*O* and *N*-sulfation reactions in heparin, combinations which allow systematic modification of the substitution patterns of heparin at I-2, A-6, and A-2 positions.

It is well-established that modification of the sulfation pattern of GAGs alters their biochemical properties. The reduction of the sulfation extent can provide benefits to their biological potential; for example, selective 6-*O*-desulfation, but not 2-*O*-desulfation, supports angiostatin activity by Hp [78]. Moreover, the *O*-desulfation process applied to Hp- and HS-like sequences increases the anti-factor Xa activity of oversulfated GAGs, sourced originally from *E. coli* [79], while *N*-sulfate and 3-*O* sulfate groups are crucial for high anticoagulant activity. Exploring the desulfation process has allowed not only analysis of the relation between the anionic groups and biological activity of GAGs, but also enabled the identification of conditions that facilitate their selective removal and the systematic study of the essentially complete substitution patterns in Hp, providing NMR assignments for these Hp-derived structures [80] (Figure 2). The desulfation of CS was first achieved using acidified methanol, proceeding via formation of the methyl ester, which was subsequently cleaved in basic conditions to generate desulfated CS [81], an approach which was also applied more recently [82].

Regioselective de-*N*-sulfation can be achieved under reaction conditions that are mild enough to avoid depolymerization [83]. De-*O*-sulfation, employing the same reagents, requires a much more prolonged reaction at higher temperatures, particularly if complete, or nearly-complete, de-*O*-sulfation is required [84]. These conditions, termed solvolytic desulfation, typically involve heating the pyridinium salt of the GAG in dimethyl sulfoxide (DMSO) in the presence of methanol or water. The method can be used to partially, or progressively, de-*O*-sulfate the polymer, avoiding the concomitant depolymerization of the chain. A similar approach was applied to oversulfated Hp to generate novel anticoagulants [85]. A systematic study, using the pyridinium salt of Hp in a solvent mixture of *N*-methylpyrrolidinone-water (NMP-H_2_O), was reported to achieve the highest selectivity of de-*O*-sulfation in glucosamine (at position 6) over iduronate (at position 2), while avoiding polymer degradation and achieving complete de-*N*-sulfation [86]. Furthermore, a recent study of Hp stability revealed that complete de-*N*-sulfation of this GAG can be achieved in a purely aqueous medium [87]. Although the aqueous approach required a highly acidic environment and a fairly high temperature (pH 1/60 °C), significant depolymerization of heparin was not detected. The drawback of this method, however, is the long reaction time (24 h) required for complete *N*-sulfate removal. The same study suggested that some level of de-*O*-sulfation selectivity is possible in acidic aqueous medium (at higher temperature of 80 °C, however, this requires further investigation.

Strategies employing a silylating agent, such as *N*-tert-butyldimethylsilyl-*N*-methyltrifluoroacetamide (MTBSTFA), were also used for regioselective de-*O*-sulfation. These agents can cleave sulfates at the 6 position selectively and convert the primary hydroxyl groups into trimethylsilyl ethers [88,89,90,91,92,93]. The specific 6-*O*-desulfation of low molecular weight Hp provided compounds with increased inhibitory activity of erythrocyte rosetting caused by the parasite *Plasmodium falciparum*, the causative agent of malaria, and exhibited attenuated anticoagulant activities [94].

Although sulfate group removal at secondary hydroxyl positions is more challenging, especially when selectivity between two secondary hydroxyl positions is required, a few cases in the literature have been reported for a fucan and galactofucan from natural sources [95,96], which suggests that some scope for this approach in GAG modification may yet exist.

In Hp and HS, a ring closure reaction that proceeds by the formation of epoxides in the iduronate 2-sulfate residue was exploited to achieve selective de-*O*-sulfation in IdoA2S residues [97,98], accompanied by some reduction in molecular weight. It is also possible to generate Hp derivatives with epoxide at these residues using milder basic conditions [98], which can then be employed for additional modifications through an attack of the epoxide. Furthermore, epoxide groups can also be opened in aqueous media [99,100].

**Figure 3:**
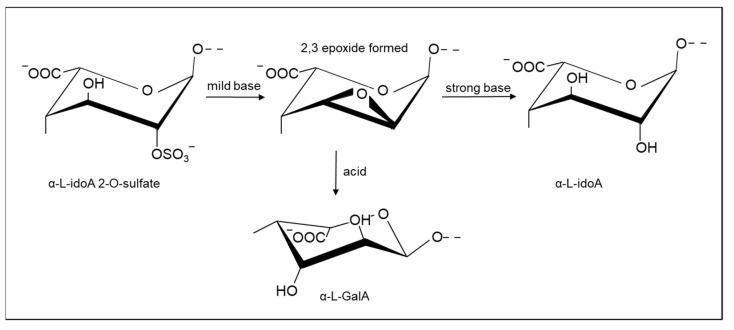
Reactions of 2-*O*-sulfated L-IdoA 2-sulfate in heparin and heparan sulfate under basic conditions and subsequent opening in acid or base. Treatment in strong base creates de-*O*-sulfated α L-iduronate residues, while treatment in acid generates α L-galacturonate residues involving inversion of the stereochemistry at positions 2 and 3.

Starting from the epoxide derivative, the selective de-*O*-sulfation of iduronate can also be achieved through additional treatment with a strong base [97]. On the other hand, by treating the epoxide heparin with acid, unnatural α L-GalA residues can be generated in which the stereochemistry at C2 and C3 is inverted with respect to α L-IdoA (Figure 3). It is possible to control the extent of epoxide formation by employing limited basic conditions, followed by epoxide ring opening in aqueous conditions, thereby permitting partial 2-de-*O*-sulfation [100].

The strong basic conditions by which the direct de-*O*-sulfation of L-IdoA 2S residues is achieved also modifies those glucosamine residues bearing both *N*- and 3-sulfation in Hp via the formation of *N*-sulfated aziridine groups [101]. A derivative of Hp-containing aziridine groups was also prepared from chemically modified (over-sulfated) Hp [102] and, by analogy with the epoxide derivative above, potentially allows for the preparation of a wide range of derivatives or subsequent modifications.

While discussing Hp modification through basic treatment, it is worth noting some additional modifications that could occur at the reducing terminus in material subjected to these conditions. For example, the alkaline hydrolysis of Hp (benzyl ester), during the reduction of *N*,6-sulfated glucosamine residues, may result in the appearance of two bicyclic structures, i.e., 1,6-anhydro glucose and mannose, located at the reducing terminus of the polymer. The base-catalyzed epimerization of reducing sugars has long been known (the Lobry de Bruyn-van Eckenstein rearrangement) and an analogous process was reported for glucosamine residue (GlcNS(6S)) [103], the basic treatment of which resulted in the appearance of 2-deoxy-6-*O*-sulfo-2-sulfamino-D-mannose. A similar reaction was reported to occur in *N*-acetylated units (GlcNAc) [104].

Controlled 2-*O*-desulfation can be combined with periodate oxidation (glycol splitting) of non-sulfated uronate residues, which induces further conformational degrees of freedom by generating rotatable bonds within the Hp chains. Since the glycol splitting reaction also modifies the essential GlcA residues at the active site for antithrombin, the products lose most of the anticoagulant activity of the original Hp, while other biological activities are either retained or modulated, depending on the extent of 2-*O*-desulfation and glycol splitting. These combined chemical modifications were employed to modulate heparin-growth factor binding and associated biological activities [105]. 

The complete 6-*O*-desulfation of intact Hp was also achieved using *N*-(tert-butyl dimethylsilyl)-*N*- methyl trifluoroacetamide (MTBSTFA). This procedure improved the enzyme digestibility of products, overcoming the disadvantage of the low digestibility of 6-*O*-desulfated Hp, generated by other procedures (silylating reagents such as (*N*, *O*-bis(trimethylsilyl)acetamide (BTSA)). The resulting enzymatic digestibility of the completely 6-*O*-desulfated Hp returned to levels similar to those observed for intact Hp [91,106]. 

### 2.2. Modification of Acetylation 

Generally, *N*-acylation derivatizations of GAGs are based on the use of anhydrides as acylating agents. *N*-acylation, most commonly *N*-acetylation, is readily achieved using acetic anhydride in mildly basic aqueous conditions, such as sodium hydrogen carbonate (reviewed; [106]) and was also applied to de-*N*-sulfated Hp using propionic and butyric anhydrides to generate the respective N-acyl derivatives [107]. Microwave irradiation was also used to form *O*-sulfated, *N*-acetylated Hp in a one-pot reaction using trimethylamine sulfur trioxide in a solvent system of trithylamine and pyridine, followed by acetic anhydride. This gave a 90% yield of the protected *O*-sulfated, *N*-acetylated material, which was then de-protected [73]. Replacing acetic anhydride with alternative acyl precursors could enable the rapid generation of other *N*-acylated heparin derivatives. *N*-hydroxysuccinimide esters in a solvent mixture of DMSO and saturated aqueous sodium bicarbonate can also be used to generate Hp products with amides bearing aromatic residues [108]. The challenge of solubilizing GAGs in suitable organic solvents has generally precluded the use of acyl chlorides as acylating agents. A further consideration is the susceptibility of *N*-sulfates to acid hydrolysis; on the other hand, their very susceptibility can be exploited to provide deliberate desulfation and allow subsequent *N*-acylation. Modifications of amino groups in HS/Hp, whether native or introduced via de-*N*-sulfation, have been exploited to prepare a variety of *N*-acyl Hp and/or partially de-*N*-sulfated Hp derivatives, followed by reaction with *N*-acylating species for the introduction of fluorescent tags, including fluorescein, coumarin, the dansyl group, and rhodamine B [4,109]. An HS analogue, modified at the principal positions of *O*-sulfation and *N*-sulfation, followed by acetylation, was found to regulate the processing of the amyloid precursor protein by the Alzheimer’s β-secretase (BACE-1) and exhibited activities against two other structurally related aspartyl proteases (cathepsin-D and renin), along with low anti-Xa activity [53].

Alternative reagents could be applied to reduce the probability of concomitant *N*-desulfation. A mild acylating method for amines, utilizing iodine catalysis, solvent-free acetic anhydride, and other acetylating agents, was proposed by Phukan et al. following the work of Field et al. on *O*-acetylation [110]. This method, which employs iodine catalysis to quantitatively *N*-acylate amines under solvent-free conditions at room temperature [111], was also used to generate fluoroacetylated derivatives of Hp for ^19^F NMR studies [112]. Ionic liquids were used for cellulose derivatization, particularly acetylation using 1-ethyl-3-methylimidazolium acetate [113]. This method could provide an approach to the *O*-acetylation of GAGs.

Other approaches for selective *O*-acylation of carbohydrates, which could be applied to generate *O*-acylated GAG derivatives [114,115], have been developed. Although such structures are not known to exist naturally, heparin and Hp fragments can be specifically *O*-acetylated by the use of tetrabutylammonium or tributylammonium salts of the anionic polysaccharides, carboxylic acid anhydrides, and 4-dimethylaminopyridine in DMF [116]. *O*-acylation, achieved by *O*-butyrylation or *O*-hexanoylation of depolymerized Hp fragments, provided products with very low anticoagulant effects in vitro, but high activity against both human immunodeficiency viruses-1 and -2 (HIV-1 and HIV-2) induced cytopathic effects, without cytotoxicity [117]. Moreover, a peptide selected from a library was used to catalyze selective *O*-acylation of glucopyranosyl derivatives at C3 (97:3 selectivity at C3 versus C4) [118]. Furthermore, high selectivity and yield were reported for organocatalytic regioselective acylation at C4 of octyl-α D-glucopyranoside. High selectivity was also obtained with chloroform as a solvent, but this required the presence of a hydrophobic substituent on the carbohydrate to ensure solubility [119]. In the case of GAGs, solubility in such non-polar solvents can be provided through the formation of organic salts, using tertiary or quaternary amines, or by previous protection.

Other acylation methods were used on variously protected monosaccharides, which potentially could be applied to GAGs. For example, *O*-acylation of 1-methyl protected galactose, using an acylating agent containing cyanide counter ions, generated an apparent preference for axial hydroxyl groups [120]. The organic solvent system employed would require tertiary or quaternary amine GAG salts to be formed prior to modification, or alternative solvents to be identified. Dibutylstannylene-mediated modifications could also be applied to GAG modification, with the prior de-sulfation in some family members a necessity to provide vicinal diols. Using dibutylstannylene for acylation was shown to be highly regioselective with high yields and reaction times around 12 h [121]. Supplementing dibutylstannylene acylation with microwave irradiation drastically reduced this reaction time to the order of 15 min. The resulting acylation occurred as a mixture of 2-*O*, 3-*O*, and 2,3-*O* modified monosaccharides, with prior primary hydroxyl protection; some unmodified carbohydrate remained when using either toluene or acetonitrile solvation [122]. 

**Figure 4:**
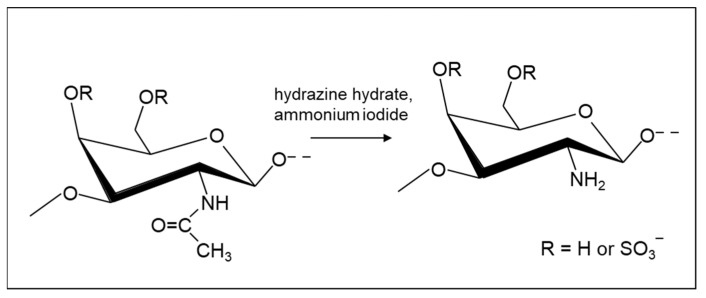
Potential selective de-*N*-acetylation of β-D-galactosamine in CS or DS achieved using hydrazine hydrate with ammonium salts as catalysts.

De-*N*-acetylation, which is a form of amide hydrolysis—a notoriously difficult reaction—is achieved conventionally by cleaving the amide bonds using harsh basic conditions with limited functional group tolerance [123] and, alongside de-*O*-sulfation, is likely to result in considerable reduction in the polysaccharide chain length. A notable advance in this regard was made by Shimizu et al., who developed ammonium salt-catalyzed hydrazinolysis of inactivated amides that is capable of cleaving *N*-acetyl groups from the amino sugar derivatives under microwave irradiation [124]. This result and preliminary work in the authors’ laboratories suggested that a similar methodology could be used to accomplish selective *N*-deacetylation of GAG structures (Figure 4). A method for the cross-linking of chitosan via free amino groups using a cross-linker was published, which may also be promising for GAGs [125]. 

Chitlac, containing a chitosan backbone to which lactitol moieties can be chemically inserted via a reductive N-alkylation with lactose [126], interacts with galectin-1, making it relevant to the production of biomaterials. Quaternization of chitosan increases its water solubility and can be achieved directly on the free amine or by introducing an acryl chain, containing a quaternary ammonium group on to the amine of chitosan, by Michael addition.

### 2.3. Oxidation and Reduction Reactions

Oxidized Hp preparations can be obtained using periodate, which acts on cis-diols, and more extensively oxidized samples can be attained by prior de-*O*-sulfation of iduronate 2-sulfate residues to generate additional cis-diols. Cleavage of the polysaccharide chain to generate fragments can be achieved by reduction, followed by acid hydrolysis [127]. It was employed to depolymerize heparin, including cleaving the antithrombin binding site [128]. Alternatively, periodate oxidation and reduction without subsequent hydrolysis, which generates stable products that maintain the molecular weight of the polysaccharide, was used to investigate the effect on anticoagulation, since the antithrombin binding site contains a GlcA residue with a susceptible cis-diol [129]. Periodate oxidation carried out on selectively de-sulfated heparin, including a graded series of N-acetylations, generates fragments termed ‘glycol-split heparins’ that maintain their ability to inhibit heparanase, but with attenuated anticoagulant activity [130]. Furthermore, an Hp derivative, obtained after exhaustive periodate oxidation, showed anti-HIV activity and a safety ratio (arbitrary units of anti-HIV activity per anticoagulant international unit) 48-fold better than standard Hp [131]. From the biotechnological point of view, these data suggest that periodate oxidation may be an interesting choice for producing Hp derivatives with low anticoagulant activity, while minimizing the loss of other biological properties.

**Figure 5:**
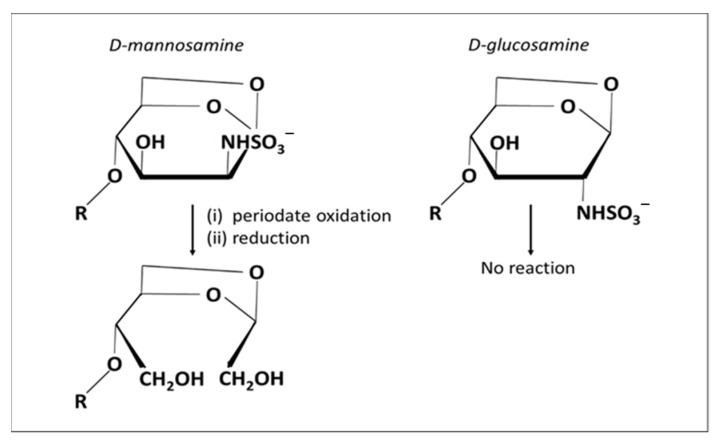
Terminal 1,6-anhydro-D-mannosamine residues (upper left) formed during depolymerization under basic conditions, during enoxaparin manufacture, are oxidized by periodate to cleave the carbon to carbon (C2-C3) bond. The aldehydes formed can be reduced further to alcohols (lower right). Terminal 1,6-anhydro-D-glucosamine residues (upper right) in enoxaparin are not susceptible to periodate oxidation.

Alekseeva et al. showed that oligosaccharides from enoxaparin (a low molecular weight Hp) can be split, not only at the level of internal non-sulfated uronate residues, but also at their reducing end amino sugar residues; in particular, at the terminal *N*-sulfated 1,6-anhydro mannosamine residues that are formed during the production of low molecular weight Hp under alkaline conditions (Figure 5) [132]. The kinetics of this process are much slower than glycol splitting of non-sulfated iduronate [133]. 

This result will be useful to optimize the preparation of glycol-split low molecular weight heparins. If oxidation of the 2,3 cis-diols of iduronate residues is required in HS/Hp, preliminary 2-de-*O* sulfation, to generate vicinal diols through an epoxide intermediate [97], allows the modification to be achieved to a higher extent, and is susceptible to further modification, such as reduction or reductive amination [100]. Heparin derivatives involving the periodate cleavage of 2,3 vicinal diols in non-sulfated uronate residues (glycol-split), and the substitution of *N*-sulphamido with *N*-acetamido groups in glucosamine residues, were found to be capable of inhibiting neutrophil elastase activity in vitro, interacting with interleukin 8 (IL-8) and tumor necrosis factor α (TNF-α), while exhibiting attenuated anticoagulant properties [134]. Glycol-split GAG derivatives could provide a platform from which improved therapeutic agents with different applications, including anti-inflammatory agents, might be developed. The vicinal 2- and 3-hydroxyl groups of the iduronate residues of 2-de-*O*-sulfated heparin can be oxidized to dialdehydes, followed by Pinnick oxidation (conversion of aldehydes to carboxylic acids by sodium chlorite in mild acid). The resulting carboxylated heparin is endowed with heparanase inhibitory and anti-tumor activities [130].

Oxidation was also reported for CS and DS polysaccharides and was directed both to the primary hydroxyl group of GalNAc, as well as periodate oxidation of vicinal diols to the corresponding aldehydes [2]. The resulting aldehyde functions can then cross-link the amino-bearing side-chains of lysine, or other amine residues, to generate non-toxic and blood-compatible hydrogels with biological applications [135,136]. The secretion of GAGs, such as CS by fibroblasts, are a crucial phase of wound healing, which allows for the formation of a hydrophilic matrix suitable for remodeling during healing. This class of hydrogels, prepared from CS-like molecules, has the potential to act as wound dressing materials [136].

Additionally, oxidation of the GalNAc primary alcohol in CS-A or -C was reported via the formation of a C-6 aldehyde on GalNAc4S, with the elimination of the sulfate group at position *O*-4 to provide α, β-unsaturation. This can be achieved by employing the 4-acetamido- (2,2,6,6-tetramethyl-1-piperidinyloxy) TEMPO radical and aqueous sodium chlorate [137]. The most widely used oxidation reaction for CS, however, is the functionalization of aldehyde in the CS backbone, generated by periodate cleavage. An example of its subsequent use is cross-linking by employing hydrazides [2]. Panagos et al. reported oxidation of a trisaccharide with a GalNAc4S reducing end to *N*-acetylgalactosaminic acid [138].

Oxidation involving TEMPO protocols were used for primary hydroxyl oxidation on GlcNAc residues in HA, amongst various carbohydrates, and have the potential for use on the primary hydroxyl groups in other GAGs. A number of methods exist using different co-catalysts with sodium hypochlorite (reviewed; [139]). One approach employed a combination of sodium hypochlorite and sodium bromide to return reduced TEMPO to the active oxoammonium ion form following carbohydrate oxidation [140]. A subsequent method developed without sodium bromide, at the increased temperature of 20 °C, maintained comparable reaction rates without losing specificity for the primary alcohol [141]. Such small molecule catalysts were also replaced by laccase enzymes to facilitate the regeneration of an oxoammonium cation during cellulose oxidation [142]. The laccase/TEMPO method has additionally been used to oxidize arabinoxylan, as well as guar galactomannan and konjac glucomannan, however, it has not yet been applied to GAGs [143]. The effect of reactive oxygen species on a range of GAGs were also investigated, but the consequences included depolymerization, de-sulfation, and oxidation [144]. 

During an investigation of acidic fibroblast growth factor (FGF-1) activity, the reduction of carboxylate groups to the primary alcohol was correlated with an impaired ability to bind to the cell surface receptor [145]. 

### 2.4. Other Modifications

#### 2.4.1. Amination 

Carbohydrate amination, or the addition of an amine group, was studied in connection with bacterial immunogens and cancer vaccines due to their ability to form direct protein-carbohydrate conjugates [146]. Their biomedical importance can also be coupled with variations in physico-chemical properties and potential applications in drug delivery and food production [147]. Native amino groups present in GAG family members, usually either *N*-sulfated or *N*-acetylated, can be converted to their unsubstituted form using the de-*N*-sulfation and de-*N*-acetylation methods reviewed above. The reductive amination of oligosaccharides, using a sodium cyanoborohydride aminating/reducing agent in aqueous buffer, has been used to prepare aminated GAG mimetics and carbohydrate precursors for protein conjugation [146,148]. An alternative amination method, previously reported for hemicellulosic xyloglucan, was performed in aqueous buffer utilizing an ethylene diamine aminating agent [147]. 

The aldehyde group at the reducing end of GAGs was often used in reductive amination to conjugate it with other molecules. Aldehydes can also be created by periodate oxidizing vicinal 2,3 hydroxyls of uronic acid in Hp, and this approach was used to connect a biotinylated compound to improve the pharmacokinetic/pharmacodynamic profile [149]. An aniline-catalyzed reductive amination, in preparation of reducing an end-clickable chito-oligosaccharide, was also reported [150].

#### 2.4.2. Phosphorylation

GAG phosphorylation was reported on the protein linker region of amino acid linked xylose residue as discussed in the introduction, yet little research has been performed on the potential phosphorylation effects throughout GAG chains. Xylose phosphorylation prevents galactosyltransferase activity during biosynthesis, but phosphorylation along the length of a GAG chain may lead to different protein interaction and physico-chemical properties [151]. It should be noted, however, that the different properties of the salts of phosphates, compared to sulfates, may render such derivatives poorly soluble, particularly their divalent cation forms. Nevertheless, numerous publications have described the phosphorylation of non-GAG carbohydrates, resulting in increased protein adsorption [152], as well as anti-viral [153] and anti-oxidant properties [154,155]; thus, further application to GAGs may be warranted.

A chitosan *O*- and *N*-phosphorylation method was outlined using a DMF-urea solvent system at high temperature, with phosphoric acid acting as the phosphorylating agent [156]. While this method provided a potential route for GAG phosphorylation, certain family members would require prior desulfation. Alternative methods, which replaced phosphoric acid with phosphorus pentoxide in methane sulfonic acid, enabled the reaction to be run at 0–5 °C without the use of organic solvents, and these methods were applied to modify both chitin and chitosan [157,158]. Additionally, microwave irradiation was applied to chitosan *N*-phosphorylation when dissolved in acetic acid with the dropwise addition of phosphoric acid, resulting in *N*-phosphorylation [159]. 

#### 2.4.3. Derivatization of Carboxylates 

The activation of the carboxylate groups of uronate residue can also be used to generate derivatized GAGs. The derivatization of carboxyl groups through the formation of activated esters, for example, using 1-ethyl-3-(3-dimethylaminopropyl) carbodiimide (EDC) derivatives and amide formation following a reaction with an amine-containing reagent (label, cross-linker, or protein), can be used. This approach is also potentially feasible for other nucleophilic functional groups, such as hydrazides or oximes (reviewed; [160]. The carboxyl group could also be activated by triazine derivatives [161] and an alternative condensing agent 4-(4,6-dimethoxy-1,3,5-triazin-2-yl)-4-methylmorpholinium chloride (DMTMM) is commercially available. D’Este et al. compared DMTMM to EDC/N-hydroxysuccinimide (NHS) activation chemistry for HA ligation using an array of substrates, including small, large, and functional molecules. For all the substrates tested, DMTMM was more efficient than EDC/NHS for ligation of amine to HA [162]. HA was conjugated with folic acid (FA) via a reduction-sensitive disulfide linkage to form a HA-grafted polymer (HA-ss-FA), which was used as a nanocarrier for a dual-CD44 and FA receptor-targeted delivery system for anti-tumor drugs [163]. 

Studies showed that hydrogels, prepared by reacting the carbodiimide/*N*-hydroxysulfosuccinimide (EDC/s-NHS)-activated carboxylic acid group of Hp with amine functionalized four-armed (star-shaped) poly(ethylene glycol) (starPEG), were able to effectively stimulate angiogenesis of human endothelial cells and the differentiation of human mesenchymal stem cells, by promoting the delivery of vascular endothelial growth factor and bone morphogenic protein-2, as well as non-covalent binding of soluble mitogens such as FGF-2 [164,165]. These materials present similar mechanical properties to the physical conditions of different tissues, ranging in elasticity from brain to osteoid tissue, presenting a valuable biotechnological resource for neurodegenerative diseases. 

It was also shown [166] that esterification of carboxylic acids can be achieved using 1-mercapto-11-undecanoic acid. The activation requires higher concentrations of carbodiimide and NHS, as well as longer reaction times, but it is more stable and less prone to form *N*-acylurea derivatives [167]. 

Toida described a method for producing alkyl-esterified GAGs [168]. The method comprised of reacting a trialkylsilyldiazoalkane with HA in DMSO and methanol. Alkyl-esterification takes place at the carboxyl groups and can be either partial or total. In another report, the methyl ester of low molecular weight hyaluronan was prepared using trimethylsilyl diazomethane [169]. Methyl esters of hyaluronate are more stable to enzymes such as hyaluronidase and methyl esterase. In addition, the hydration properties of the new compounds are more favorable than the native HA [169] and, for this reason, are employed in cosmetics.

#### 2.4.4. Derivatization of Hydroxyl Groups

The alkylation in water of the *O*-3 and *O*-6 positions of Hp GlcN units in the presence of NaOH, with (3-chloro-2-hydroxypropyl) trimethylammonium chloride as the alkylating agent, was reported [170]. These conditions allowed etherification of such positions with the attachment of the cationic trimethylammonium-2-hydroxypropyl (TMAHP) spacer. Alkylation could also be used to increase the solubility of the GAG polymer in organic solvents. Highly soluble carboxymethyl chitosan was obtained through an alkylation process, which employed monochloroacetic acid and sodium hydroxide under optimized conditions [171]. The synthesis of alkylated and fluoroalkylated semi-synthetic GAGs are useful in a number of therapeutic and cosmetic applications and for the potential treatment of inflammatory diseases and skin disorders. Several pharmaceutical compositions containing alkylated semi-synthetic GAGs are currently used to treat inflammatory degenerative diseases [172,173,174] making this modification an important tool for the pharmaceutical industry.

*O*-methylation was applied to sulfated GAGs. Standard methylation procedures employ methanol and sodium hydroxide [175]. Recent refinements to the analysis of GAG oligosaccharides, which presents challenging solubility in DMSO, include those introduced by Huang, Pomin and Sharp, who proposed using reduced CS hexasaccharides, converted to their triethylamine (TEA) salts, resuspended in anhydrous NaOH in DMSO, followed by the addition of iodomethane [176]. Hu and Borges also created an efficient procedure for the permethylation of glycans, by again using DMSO and iodomethane, but it relied on testing the water content during the procedure [177]. An alternative, rapid iodomethane-based methylation method for carbohydrates enabled permethylation in as little as 6 min, depending on the base and solvent system used, with high yields [178]. Methylated derivatives are particularly useful for glycosyl-linkage characterization, with linkage positions being determined from monosaccharide GC-MS retention times and mass spectrometry [179]. This approach could be invaluable if applied to the characterization of novel GAGs with unusual or hybrid structures.

Two classes of phenolic compounds with potential pharmaceutical applications, flavonoids and gallates, include a wide variety of functional groups that could be used for GAG modification. These derivatives have exhibited anti-microbial and anti-cancer properties, alongside apoptosis induction and lipid membrane damage [180,181,182]. Tri-*N*-methyl chitosan *O*-modification via gallic and caffeic acid addition were also reported, involving a prior step to transform these modifying groups into their chloride form. The products of gallic and caffeic acid grafted onto tri-*N*-methyl chitosan resulted in degrees of substitution of 0.75 and 0.72, respectively [183]. Reactions in aqueous solvents were also used to generate carbohydrate *O*-modification via flavonoid addition when employing ascorbic acid and hydrogen peroxide as an initiator system [184]. 

A method similar to that described for microwave irradiation assisted carbohydrate acylation was described for the regioselective alkylation of galactosides. This method could be applied to GAGs, however, the requirement of a vicinal diol to facilitate dibutylstannylene interaction necessitates prior de-sulfation if used for certain GAG family members. Using this microwave assisted protocol, alkylated products can be generated with yields ranging from 45–86%, depending on the compound added, within 20 min [185]. Additionally, treatment of CS with PEG-diglycidyl ether in aqueous NaOH generated CS cross-linking hydrogels that were evaluated as drug-delivery vehicles. This reaction proceeded by a non-regioselective attack of both GalNAc and GlcA hydroxy groups of CS-A and C on the epoxide rings of PEG-diglycidyl ether [60]. 

## 3. Concluding Remarks

Following any GAG modification, it is essential to be able to assess the extent and position of the structural change and, to do so, the preeminent method remains NMR spectroscopy. Extensive data exists for several GAGs, including CS [186,187,188] and Hp/HS [80,189]. Owing to their ability to modulate many biological functions, the breadth of available structural modifications for carbohydrates has expanded and here, alongside more established techniques, some less conventional reactions have also been reported for polysaccharide derivatization that may have future application to GAGs. 

When interpreting the results of investigations of biological activity employing modified GAGs, it is also important to consider the potentially complex changes in the conformation of the individual residues, particularly iduronate, altered glycosidic bond angles, and the resulting overall polysaccharide characteristics [21]. These features are difficult to assess and lie at the forefront of current spectroscopic and molecular simulation research into the GAGs. 

A wide range of biological activities have been investigated using modified GAGs, but this number is likely to expand rapidly in the near future. Their scientific usefulness was highlighted in recent investigations on the role of GAGs in viral infection [54] and their potential to be applied to numerous medical and biotechnological challenges is becoming the focus of renewed attention [1]. 

From this survey, several recent advances are worth highlighting: the improved methods for the removal of *N*-acetyl groups, cleaner and more reliable sulfation procedures, and an improved understanding of some of the conformational consequences of GAG modifications that are emerging from detailed spectroscopic investigations will all enable progress to be made. Chemically modified GAGs have established themselves as a valuable component of the experimentalist’s arsenal with which to investigate GAG-protein interactions and biological activities, and are set to continue to make valuable contributions that complement parallel advances in protein structural prediction and bioinformatics approaches. 

## Data Availability

Not applicable.

## References

[1] DeAngelis P.L. (2012). Glycosaminoglycan polysaccharide biosynthesis and production: Today and tomorrow. Appl. Microbiol. Biotechnol..

[2] Bedini E., Lavezzi A., Iadonisi A. (2016). Chemical Derivatization of Sulfated Glycosaminoglycans. Eur. J. Org. Chem..

[3] Karst N.A., Linhardt R.J. (2003). Recent Chemical and Enzymatic Approaches to the Synthesis of Glycosaminoglycan Oligosaccharides. Curr. Med. Chem..

[4] Fernández C., Hattan C.M., Kerns R.J. (2006). Semi-synthetic heparin derivatives: Chemical modifications of heparin beyond chain length, sulfate substitution pattern and *N*-sulfo/*N*-acetyl groups. Carbohydr. Res..

[5] Linhardt R.J., Dordick J.S., Deangelis P.L., Liu J. (2007). Enzymatic synthesis of glycosaminoglycan heparin. Semin. Thromb. Hemost..

[6] Vibert A., Lopin-Bon C., Jacquinet J.-C. (2011). Efficient and Stereocontrolled Construction of Homo- and Heterogeneously 4- and 6-Sulfated Biotinylated Chondroitin Oligomers. Eur. J. Org. Chem..

[7] Guimond S.E., Turnbull J.E., Yates E.A. (2006). Engineered bio-active polysaccharides from heparin. Macromol. Biosci..

[8] Pavão M.S., Mourao P.A., Mulloy B., Tollefsen D.M. (1995). A unique dermatan sulfate-like glycosaminoglycan from ascidian. Its structure and the effect of its unusual sulfation pattern on anticoagulant activity. J. Biol. Chem..

[9] Ernst S., Langer R., Cooney C.L., Sasisekharan R. (1995). Enzymatic degradation of glycosaminoglycans. Crit. Rev. Biochem. Mol. Biol..

[10] Volpi N. (2006). Therapeutic Applications of Glycosaminoglycans. Curr. Med. Chem..

[11] Dietrich C.P., Nader H.B., Straus A.H. (1983). Structural Differences of Heparan Sulfates According to the Tissue and Species of Origin. Biochem. Biophys. Res. Commun..

[12] Sasisekharan R., Venkataraman G. (2000). Heparin and heparan sulfate: Biosynthesis, structure, and function. Curr. Opin. Struct. Biol..

[13] Gandhi N.S., Mancera R.L. (2008). The structure of glycosaminoglycans and their interactions with proteins. Chem. Biol. Drug Des..

[14] Hopwood J.J., Robinson H.C. (1973). The Molecular-weight distribution of glycosaminoglycans. Biochem. J..

[15] Thompson L.D., Pantoliano M.W., Springer B.A. (1994). Energetic Characterization of the Basic Fibroblast Growth Factor-Heparin Interaction: Identification of the Heparin Binding Domain. Biochemistry.

[16] Yang C., Cao M., Liu H., He Y., Xu J., Du Y., Liu Y., Wang W., Cui L., Hu J. (2012). The high and low molecular weight forms of hyaluronan have distinct effects on CD44 clustering. J. Biol. Chem..

[17] Gama C.I., Tully S.E., Sotogaku N., Clark P.M., Rawat M., Vaidehi N., Goddard III W.A., Nishi A., Hsieh-Wilson L.C. (2006). Sulfation patterns of glycosaminoglycans encode molecular recognition and activity. Nat. Chem. Biol..

[18] Swarup S., Huang W., Mackay T.F., Anholt R.R. (2013). Analysis of natural variation reveals neurogenetic networks for Drosophila olfactory behavior. Proc. Natl. Acad. Sci. USA.

[19] Martín F.A., Murphy R.P., Cummins P.M. (2013). Thrombomodulin and the vascular endothelium: Insights into functional, regulatory, and therapeutic aspects. Am. J. Physiol.-Heart Circ. Physiol..

[20] Rudd T.R., Yates E.A. (2012). A highly efficient tree structure for the biosynthesis of heparan sulfate accounts for the commonly observed disaccharides and suggests a mechanism for domain synthesis. Mol. BioSystems.

[21] Meneghetti M.C., Hughes A.J., Rudd T.R., Nader H.B., Powell A.K., Yates E.A., Lima M.A. (2015). Heparan sulfate and heparin interactions with proteins. J. R. Soc. Interface.

[22] Esko J.D., Zhang L. (1996). Influence of core protein sequence on glycosaminoglycan assembly. Curr. Opin. Struct. Biol..

[23] Malmstrom A., Bartolini B., Thelin M.A., Pacheco B., Maccarana M. (2012). Iduronic acid in chondroitin/dermatan sulfate: Biosynthesis and biological function. J. Histochem. Cytochem..

[24] Sugahara K., Kitagawa H. (2000). Recent advances in the study of the biosynthesis and functions of sulfated glycosaminoglycans. Curr. Opin. Struct. Biol..

[25] Raman R., Sasisekharan V., Sasisekharan R. (2005). Structural insights into biological roles of protein-glycosaminoglycan interactions. Chem. Biol..

[26] Meneghetti M.C.Z., Gesteira Ferreira T., Tashima A.K., Chavante S.F., Yates E.A., Liu J., Nader H.B., Lima M.A. (2017). Insights into the role of 3-*O*-sulfotransferase in heparan sulfate biosynthesis. Org. Biomol. Chem..

[27] Ori A., Free P., Courty J., Wilkinson M.C., Fernig D.G. (2009). Identification of heparin-binding sites in proteins by selective labeling. Mol. Cell. Proteom..

[28] Xu R., Ori A., Rudd T.R., Uniewicz K.A., Ahmed Y.A., Guimond S.E., Siligardi G., Yates E.A., Fernig D.G. (2012). Diversification of the structural determinants of fibroblast growth factor-heparin interactions: Implications for binding specificity. J. Biol. Chem..

[29] Li Y., Sun C., Yates E.A., Jiang C., Wilkinson M.C., Fernig D.G. (2016). Heparin binding preference and structures in the fibroblast growth factor family parallel their evolutionary diversification. Open Biol..

[30] Thao B.P. (2019). Selective Labelling of Arginine Residues in Protein Sulfated Glycosaminoglycan Binding Sites. Ph.D. Thesis.

[31] Hricovini M., Hricovini M. (2018). Solution Conformation of Heparin Tetrasaccharide. DFT Analysis of Structure and Spin(-)Spin Coupling Constants. Molecules.

[32] Nader H.B., Dietrich C.P., Buonassisi V., Colburn P. (1987). Heparin sequences in the heparan sulfate chains of an endothelial cell proteoglycan. Proc. Natl. Acad. Sci. USA.

[33] Itakura E., Chiba M., Murata T., Matsuura A. (2020). Heparan sulfate is a clearance receptor for aberrant extracellular proteins. J. Cell Biol..

[34] Pumphrey C.Y., Theus A.M., Li S., Parrish R.S., Sanderson R.D. (2002). Neoglycans, Carbodiimide-modified Glycosaminoglycans: A New Class of Anticancer Agents That Inhibit Cancer Cell Proliferation and Induce Apoptosis. Cancer Res..

[35] Yamada S., Sugahara K. (2008). Potential Therapeutic Application of Chondroitin Sulfate/Dermatan Sulfate. Curr. Drug Discov. Technol..

[36] Murugesan S., Wiencek J.M., Ren R.X., Linhardt R.J. (2006). Benzoate-based room temperature ionic liquids—Thermal properties and glycosaminoglycan dissolution. Carbohydr. Polym..

[37] Laremore T.N., Murugesan S., Park T.-J., Avci F.Y., Zagorevski D.V., Linhardt R.J. (2006). Matrix-assisted laser desorption/ionisation mass spectrometric analysis of uncom-plexed highly sulfated oligosaccharides using ionic liquid matrices. Anal. Chem..

[38] Laremore T.N., Zhang F., Linhardt R.J. (2007). Ionic liquid matrix for direct UV-MALDI-TOF-MS analysis of dermatan sulfate and chondroitin sulfate oligosaccharides. Anal. Chem..

[39] Przybylski C., Gonnet F., Bonnaffe D., Hersant Y., Lortat-Jacob H., Daniel R. (2010). HABA-based ionic liquid matrices for UV-MALDI-MS analysis of heparin and heparan sulfate oligosaccharides. Glycobiology.

[40] Wang J.K., Luo B., Guneta V., Li L., Foo S.E.M., Dai Y., Tan T.T.Y., Tan N.S., Choong C., Wong M.T.C. (2017). Supercritical carbon dioxide extracted extracellular matrix material from adipose tissue. Mater. Sci. Eng. C Mater. Biol. Appl..

[41] Wang F., Zhao G., Lang X., Li J.P., Li X. (2017). Lipase-catalyzed synthesis of long-chain cellulose esters using ionic liquid mixtures as reaction media. J. Chem. Technol. Biotechnol..

[42] Bayón C., Cortés Á., Berenguer J., Hernáiz M.J. (2013). Highly efficient enzymatic synthesis of Galβ-(1→3)-GalNAc and Galβ-(1→3)-GlcNAc in ionic liquids. Tetrahedron.

[43] Zhang Q., Zhang S., Deng Y. (2011). Recent advances in ionic liquid catalysis. Green Chem..

[44] Zakrzewska M.E., Bogel-Łukasik E., Bogel-Łukasik R. (2010). Solubility of Carbohydrates in Ionic Liquids. Energy Fuels.

[45] Prasad K., Kaneko Y., Kadokawa J. (2009). Novel gelling systems of kappa-, iota- and lambda-carrageenans and their composite gels with cellulose using ionic liquid. Macromol. Biosci..

[46] Gericke M., Fardim P., Heinze T. (2002). Ionic liquids--promising but challenging solvents for homogeneous derivatization of cellulose. Molecules.

[47] Olivier-Bourbigou H., Magna L., Morvan D. (2010). Ionic liquids and catalysis: Recent progress from knowledge to applications. Appl. Catal. A Gen..

[48] Camp J.E. (2018). Bio-available Solvent Cyrene: Synthesis, Derivatization, and Applications. ChemSusChem.

[49] Bousfield T.W., Pearce K.P.R., Nyamini S.B., Angelis-Dimakis A., Camp J.E. (2019). Synthesis of amides from acid chlorides and amines in the bio-based solvent Cyrene™. Green Chem..

[50] Zhang J., White G.B., Ryan M.D., Hunt A.J., Katz M.J. (2016). Dihydrolevoglucosenone (Cyrene) As a Green Alternative to *N, N*-Dimethylformamide (DMF) in MOF Synthesis. ACS Sustain. Chem. Eng..

[51] Caputo H.E., Straub J.E., Grinstaff M.W. (2019). Design, synthesis, and biomedical applications of synthetic sulphated polysaccharides. Chem. Soc. Rev..

[52] Appleyard R.C., Burkhardt D., Ghosh P., Read R., Cake M., Swain M.V., Murrell G.A. (2003). Topographical analysis of the structural, biochemical and dynamic biomechanical properties of cartilage in an ovine model of osteoarthritis. Osteoarthr. Cartil..

[53] Patey S.J., Edwards E.A., Yates E.A., Turnbull J.E. (2006). Heparin Derivatives as Inhibitors of BACE-1, the Alzheimer’s a-Secretase, with Reduced Activity against Factor Xa and Other Proteases. J. Med. Chem..

[54] Mycroft-West C.J., Su D., Pagani I., Rudd T.R., Elli S., Gandhi N.S., Guimond S.E., Miller G.J., Meneghetti M.C.Z., Nader H.B. (2020). Heparin inhibits cellular invasion by SARS-CoV-2. Thromb. Haemost..

[55] Bedini E., Laezza A., Parrilli M., Iadonisi A. (2017). A review of chemical methods for the selective sulfation and desulfation of polysaccharides. Carbohydr. Polym..

[56] Hintze V., Miron A., Moeller S., Schnabelrauch M., Wiesmann H.P., Worch H., Scharnweber D. (2012). Sulfated hyaluronan and chondroitin sulfate derivatives interact differently with human transforming growth factor-beta1 (TGF-beta1). Acta Biomater..

[57] Casu B., Grazioli G., Razi N., Guerrini M., Naggi A., Torri G., Oreste P., Tursi T., Zoppetti G., Lindahl U. (1994). Heparin-like compounds prepared by chemical modification of capsular polysaccharide K5. Carbohydr. Res..

[58] Gilbert E.E. (1962). The reactions of sulfur trioxide, and its adducts, with organic compounds. Chem. Rev..

[59] Ogamo A., Metori A., Uchiyama H., Nagasawa K. (1989). Reactivity toward Chemical Sulfation of Hydroxyl Groups of Heparin. Carbohydr. Res..

[60] Zhao L., Liu M., Wang J., Zhai G. (2015). Chondroitin sulfate-based nanocarriers for drug/gene delivery. Carbohydr. Polym..

[61] Wang J., Yang W., Yang T., Zhang X., Zuo Y., Tian J., Yao J., Zhang J., Lei Z. (2015). Catalytic synthesis of sulfated polysaccharides I: Characterization of chemical structure. Int. J. Biol. Macromol..

[62] Bacilieri M., Naggi A., Ceol M., Schleicher E.D., Tosetto E., Comoli M., Torri G., Moro S., Palumbo M., Gambaro G. (2011). Inhibitory effects of glycosaminoglycans on basal and stimulated transforming growth factor-beta1 expression in mesangial cells: Biochemical and structural considerations. Glycobiology.

[63] Naggi A., Torri G., Casu B., Pangrazzi J., Abbadini M., Zametta M., Donati M.B., Lansen J., Maffrand J.P. (1987). “Supersulfated” Heparin Fragments, A New Type of Low-Molecular Weight Heparin. Biochem. Pharmacol..

[64] Liu H., Zhang Z., Linhardt R.J. (2009). Lessons learned from the contamination of heparin. Nat. Prod. Rep..

[65] Ramacciotti E., Clark M., Sadeghi N., Hoppensteadt D., Thethi I., Gomes M., Fareed J. (2011). Review: Contaminants in heparin: Review of the literature, molecular profiling, and clinical implications. Clin. Appl. Thromb./Hemost..

[66] Chen T., Li B., Li Y., Zhao C., Shen J., Zhang H. (2011). Catalytic synthesis and antitumor activities of sulfated polysaccharide from Gynostemma pentaphyllum Makino. Carbohydr. Polym..

[67] Chopin N., Sinquin C., Ratiskol J., Zykwinska A., Weiss P., Cerantola S., Le Bideau J., Colliec-Jouault S. (2015). A Direct Sulfation Process of a Marine Polysaccharide in Ionic Liquid. BioMed Res. Int..

[68] Merceron C., Portron S., Vignes-Colombeix C., Rederstorff E., Masson M., Lesoeur J., Sourice S., Sinquin C., Colliec-Jouault S., Weiss P. (2012). Pharmacological modulation of human mesenchymal stem cell chondrogenesis by a chemically oversulfated polysaccharide of marine origin: Potential application to cartilage regenerative medicine. Stem Cells.

[69] Zoppetti G., Oreste P. (2004). Process for the Preparation of Chondroitin Sulfates from K4 Polysaccharide and Obtained Products. United States Patent.

[70] Laezza A., De Castro C., Parrilli M., Bedini E. (2014). Inter vs. intraglycosidic acetal linkages control sulfation pattern in semi-synthetic chondroitin sulfate. Carbohydr. Polym..

[71] de Araújo C.A., Noseda M.D., Cipriani T.R., Goncalves A.G., Duarte M.E., Ducatti D.R. (2013). Selective sulfation of carrageenans and the influence of sulfate regiochemistry on anticoagulant properties. Carbohydr. Polym..

[72] Maza S., de Paz J.L., Nieto P.M. (2011). Microwave-assisted sulfonation of heparin oligosaccharides. Tetrahedron Lett..

[73] Xu P., Laval S., Guo Z., Yu B. (2016). Microwave-assisted simultaneous O, N-sulfonation in the synthesis of heparin-like oligosaccharides. Org. Chem. Front..

[74] Naderi A., Koschella A., Heinze T., Shih K.C., Nieh M.P., Pfeifer A., Chang C.C., Erlandsson J. (2017). Sulfoethylated nanofibrillated cellulose: Production and properties. Carbohydr. Polym..

[75] Zhang K., Brendler E., Gebauer K., Gruner M., Fischer S. (2011). Synthesis and characterization of low sulfoethylated cellulose. Carbohydr. Polym..

[76] Gill D.M., Male L., Jones A.M. (2019). Sulfation made simple: A strategy for synthesising sulfated molecules. Chem. Commun..

[77] Benedetti A.M., Gill D.M., Tsang C.W., Jones A.M. (2020). Chemical Methods for N- and O-Sulfation of Small Molecules, Amino Acids and Peptides. ChemBioChem.

[78] Lundin L., Larsson H., Kreuger J., Kanda S., Lindahl U., Salmivirta M., Claesson-Welsh L. (2000). Selectively desulfated heparin inhibits fibroblast growth factor-induced mitogenicity and angiogenesis. J. Biol. Chem..

[79] Naggi A., Torri G., Casu B., Oreste P., Zoppetti G., Li J.P., Lindahl U. (2001). Toward a biotechnological heparin through combined chemical and enzymatic modification of the Escherichia coli K5 polysaccharide. Semin. Thromb. Hemost..

[80] Yates E.A., Santini F., Guerrini M., Naggi A., Torri G., Casu B. (1996). 1H and 13C NMR spectral assignments of the major sequences of twelve systematically modified heparin derivatives. Carbohydr. Res..

[81] Kantor T.G., Schubert M. (1975). A Method for the Desulfation of Chondroitin Sulfate. J. Am. Chem. Soc..

[82] Lim J.J., Temenoff J.S. (2013). The effect of desulfation of chondroitin sulfate on interactions with positively charged growth factors and upregulation of cartilaginous markers in encapsulated MSCs. Biomaterials.

[83] Inoue Y., Nagasawa K. (1976). Selective N-Desulfation of Heparin with Dimethyl Sulfoxide containing Water or Methanol. Carbohydr. Res..

[84] Nagasawa K., Inoue Y., Kamata T. (1977). Solvolytic Desulfation of Glycosaminoglyuronan Sulfates with Dimethyl Sulfoxide Containing Water or Methanol. Carbohydr. Res..

[85] Naggi A., De Cristofano B., Bisio A., Torri G., Casu B. (2001). Generation of anti-factor Xa active, 3-*O*-sulfated glucosamine-rich sequences by controlled desulfation of oversulfated heparins. Carbohydr. Res..

[86] Baumann H., Scheen H., Huppertz B., Keller R. (1998). Novel regio- and stereoselective *O*-6-desulfation of the glucosamine moiety of heparin with N-methylpyrrolidinone-water or *N*, *N*-dimethylformamide-water mixtures. Carbohydr. Res..

[87] Kozlowski A.M., Yates E.A., Roubroeks J.P., Tommeraas K., Smith A.M., Morris G.A. (2021). Hydrolytic Degradation of Heparin in Acidic Environments: Nuclear Magnetic Resonance Reveals Details of Selective Desulfation. ACS Appl. Mater. Interfaces.

[88] Baumann H., Faust V. (2001). Concepts for improved regioselective placement of *O*-sulfo, *N*-sulfo, *N*-acetyl, and *N*-carboxymethyl groups in chitosan derivatives. Carbohydr. Res..

[89] Becher J., Möller S., Weiss D., Schiller J., Schnabelrauch M. (2010). Synthesis of New Regioselectively Sulfated Hyaluronans for Biomedical Application. Macromol. Symp..

[90] Chaidedgumjorn A., Suzuki A., Toyoda H., Toida T., Imanari T., Linhardt R.J. (2002). Conductivity detection for molecular mass estimation of per-*O*-sulfonated glycosaminoglycans separated by high-performance size-exclusion chromatography. J. Chromatogr. A.

[91] Kariya Y., Kyogashima M., Suzuki K., Isomura T., Sakamoto T., Horie K., Ishihara M., Takano R., Kamei K., Hara S. (2000). Preparation of completely 6-*O*-desulfated heparin and its ability to enhance activity of basic fibroblast growth factor. J. Biol. Chem..

[92] Matsuo M., Takano R., Kamei-Hayashi K., Hara S. (1993). A novel regioselective desulfation of polysaccharide sulfates: Specific 6-*O*-desulfation with *N*,*O*-bis(trimethylsilyl)acetamide. Carbohydr. Res..

[93] Takano R., Kanda T., Hayashi K., Yoshida K., Hara S. (1995). Desulfation of Sulfated Carbohydrates Mediated by Silylating Reagents. J. Carbohydr. Chem..

[94] Skidmore M.A., Dumax-Vorzet A.F., Guimond S.E., Rudd T.R., Edwards E.A., Turnbull J.E., Craig A.G., Yates E.A. (2008). Disruption of Rosetting in Plasmodium falciparum Malaria with Chemically Modified Heparin and Low Molecular Weight Derivatives Possessing Reduced Anticoagulant and Other Serine Protease Inhibition Activities. J. Med. Chem..

[95] Pomin V.H., Valente A.P., Pereira M.S., Mourao P.A. (2005). Mild acid hydrolysis of sulfated fucans: A selective 2-desulfation reaction and an alternative approach for preparing tailored sulfated oligosaccharides. Glycobiology.

[96] Shevchenko N.M., Anastyuk S.D., Menshova R.V., Vishchuk O.S., Isakov V.I., Zadorozhny P.A., Sikorskaya T.V., Zvyagintseva T.N. (2015). Further studies on structure of fucoidan from brown alga Saccharina gurjanovae. Carbohydr. Polym..

[97] Jaseja M., Rej R.N., Sauriol F., Perlin A.S. (1989). Novel regio- and stereoselective modifications of heparin in alkaline solution. Nuclear magnetic resonance spectroscopic evidence. Can. J. Chem..

[98] Piani S., Casu B., Marchi E.G., Torri G., Ungarelli F. (1993). Alkali-Induced Optical Rotation Changes in Heparins and Heparan Sulfates, and Their Relation to Iduronic Acid-Containing Sequences. J. Carbohydr. Chem..

[99] Wang Z., Cui Y.-T., Xu Z.-B., Qu J. (2008). Hot Water-Promoted Ring-Opening of Epoxides and Aziridines by Water and Other Nucleopliles. J. Org. Chem..

[100] Casu B., Guerrini M., Guglieri S., Naggi A., Perez M., Torri G., Cassinelli G., Robatti D., Carminati P., Giannini G. (2004). Undersulfated and Glycol-Split Heparins Endowed with Antiangiogenic Activity. J. Med. Chem..

[101] Santini F., Bisio A., Guerrini M., Yates E.A. (1997). Modifications under basic conditions of the minor sequences of heparin containing 2,3 or 2,3,6 sulfated D-glucosamine residues. Carbohydr. Res..

[102] Yates E.A., Santini F., Bisio A., Cosentino C. (1997). Evidence for a heparin derivative containing an *N*-sulfated aziridine ring that retains high anti-factor Xa activity. Carbohydr. Res..

[103] Yamada S., Watanabe M., Sugahara K. (1998). Covnersion of *N*-sulfated glucosamine to *N*-sulfated mannosamine in an unsaturated heparin disaccharide by non-enzymatic, base-catalyzed C-2 epimerization during enzymatic oligosaccharide preparation. Carbohydr. Res..

[104] Toida T., Vlahov I.R., Smith A.E., Hileman R.E., Linhardt R.J. (1996). C-2 Epimerization of *N*-Acetylglucosamine in an Oligosaccharide Derived from Heparan Sulfate. J. Carbohydr. Chem..

[105] Pisano C., Cervoni M.L., Chiarucci I. Antiangiogenic and Antitumoral Activity of Novel Heparin Derivatives Devoid of Anticoagulant Effects. Proceedings of the National Cancer Institute-European Organization for Research and Treatment of Cancer-American Association for Cancer Research Symposium (NCI-EORTC-AACR).

[106] Naik S., Bhattacharjya G., Kavala V.R., Patel B.K. (2004). Mild and eco-firendly chemoselective acylation of amines in aqueous medium. ARKIVOC.

[107] Patey S.J., Edwards E.A., Yates E.A., Turnbull J.E. (2008). Engineered heparins: Novel beta-secretase inhibitors as potential Alzheimer’s disease therapeutics. Neurodegener. Dis..

[108] Huang L., Kerns R.J. (2006). Diversity-oriented chemical modification of heparin: Identification of charge-reduced N-acyl heparin derivatives having increased selectivity for heparin-binding proteins. Bioorg. Med. Chem..

[109] Sun W., Bandmann H., Schrader T. (2007). A fluorescent polymeric heparin sensor. Chem. A Eur. J..

[110] Mukhopadhyay B., Ravindranathan-Kartha K.P., Russell D.A., Field R.A. (2004). Streamlined Synthesis of Per-*O*-acetylated Sugars, Glycosyl Iodides, or Thioglycosides from Unprotected Reducing Sugars. J. Org. Chem..

[111] Phukan K., Ganguly M., Devi N. (2009). Mild and Useful Method for N-Acylation of Amines. Synth. Commun..

[112] Lima M.A., Cavalheiro R.P., Viana G.M., Meneghetti M.C.Z., Rudd T.R., Skidmore M.A., Powell A.K., Yates E.A. (2017). 19F-labelled glycosaminoglycan probes for solution NMR and non-linear (CARS) microscopy. Glycoconj. J..

[113] Köhler S., Liebert T., Schöbitz M., Schaller J., Meister F., Günther W., Heinze T. (2007). Interactions of Ionic Liquids with Polysaccharides 1. Unexpected Acetylation of Cellulose with 1-Ethyl-3-methylimidazolium Acetate. Macromol. Rapid Commun..

[114] Zhang Z., Jin F., Wu Z., Jin J., Li F., Wang Y., Wang Z., Tang S., Wu C., Wang Y. (2017). O-acylation of chitosan nanofibers by short-chain and long-chain fatty acids. Carbohydr. Polym..

[115] Gao N., Wu M., Liu S., Lian W., Li Z., Zhao J. (2012). Preparation and characterization of O-acylated fucosylated chondroitin sulfate from sea cucumber. Mar. Drugs.

[116] Petitou M., Coudert C., Level M., Lormeau J.-C., Zuber M., Simenel C., Fournier J.-P., Choay J. (1992). Selectively O-acylated glycosamionglycan derivatives. Carbohydr. Res..

[117] Bârzu T., Level M., Petitou M., Lormeau J.-C., Choay J. (1993). Preparation and Anti-HIV Activity of O-Acylated Heparin and Dermatan Sulfate Derivatives with Low Anticoagulant Effect. J. Med. Chem..

[118] Jäger M., Minnaard A.J. (2016). Regioselective modification of unprotected glycosides. Chem. Commun..

[119] Takeuchi H., Mishiro K., Ueda Y., Fujimori Y., Furuta T., Kawabata T. (2015). Total Synthesis of Ellagitannins through Regioselective Sequential Functionalization of Unprotected Glucose. Angew. Chem. Int. Ed..

[120] Peng P., Linseis M., Winter R.F., Schmidt R.R. (2016). Regioselective Acylation of Diols and Triols: The Cyanide Effect. J. Am. Chem. Soc..

[121] Peri F., Cipolla L., Nicotra F. (2000). Tin-mediated regioselective acylation of unprotected sugars on solid phase. Tetrahedron Lett..

[122] Herradón B., Morcuende A., Valverde S. (1995). Microwave Accelerated Organic Transformations: Dibutylstannylene Acetal Mediated Selective Acylation of Polyols and Amino Alcohols using Catalytic A Mounts of Dibutyltin Oxide. Influence of the Solvent and the Power Output on the Selectivity. Synlett.

[123] Sultane P.R., Mete T.B., Bhat R.G. (2014). Chemoselective *N*-deacetylation under mild conditions. Org. Biomol. Chem..

[124] Shimizu Y., Noshita M., Mukai Y., Morimoto H., Ohshima T. (2014). Cleavage of unactivated amide bonds by ammonium salt-accelerated hydrazinolysis. Chem. Commun..

[125] Welsh E.R., Schauer C.L., Qadri S.B., Price R.R. (2002). Chitosan Cross-Linking with a Water-Soluble, Blocked Diisocyanate. 1. Solid State. Biomacromolecules.

[126] D’Amelio N., Esteban C., Coslovi A., Feruglio L., Uggeri F., Villegas M., Benegas J., Paoletti S., Donati I. (2013). Insight into the molecular properties of Chitlac, a chitosan derivative for tissue engineering. J. Phys. Chem. B.

[127] Fransson L.-Å. (1987). Periodate Oxidation of the D-Glucuronic Acid Resodies in Heparan Sulphate and Heparin. Carbohydr. Res..

[128] Islam T., Butler M., Sikkander S.A., Toida T., Linhardt R.J. (2002). Further evidence that periodate cleavage of heparin occurs primarily through the antithrombin binding site. Carbohydr. Res..

[129] Casu B., Diamantini G., Fedeli G., Mantovani M., Oreste P., Pescador R., Porta R., Prino G., Torri G., Zoppetti G. (1986). Retention of antilipemic activity by periodate-oxidized non-anticoagulant heparins. Arzneimittelforschung.

[130] Naggi A., Casu B., Perez M., Torri G., Cassinelli G., Penco S., Pisano C., Giannini G., Ishai-Michaeli R., Vlodavsky I. (2005). Modulation of the heparanase-inhibiting activity of heparin through selective desulfation, graded N-acetylation, and glycol splitting. J. Biol. Chem..

[131] Lopalco L., Ciccomascolo F., Lanza P., Zoppetti G., Caramazza I., Leoni F., Beratta A., Siccardi A.G. (1994). Anti-HIV Type 1 Properties of Chemically Modified Heparins with Diminished Anticoagulant Activity. AIDS Res. Hum. Retrovir..

[132] Alekseeva A., Casu B., Torri G., Pierro S., Naggi A. (2013). Profiling glycol-split heparins by high-performance liquid chromatography/mass spectrometry analysis of their heparinase-generated oligosaccharides. Anal. Biochem..

[133] Alekseeva A., Elli S., Cosentino C., Torri G., Naggi A. (2014). Susceptibility of enoxaparin reducing end amino sugars to periodate oxidation. Carbohydr. Res..

[134] Veraldi N., Hughes A.J., Rudd T.R., Thomas H.B., Edwards S.W., Hadfield L., Skidmore M.A., Siligardi G., Cosentino C., Shute J.K. (2015). Heparin derivatives for the targeting of multiple activities in the inflammatory response. Carbohydr. Polym..

[135] Bernhard J.C., Panitch A. (2012). Synthesis and characterization of an aggrecan mimic. Acta Biomater..

[136] Dawlee S., Sugandhi A., Balakrishnan B., Labarre D., Jayakrishnan A. (2005). Oxidized Chondroitin Sulfate-Cross-Linked Gelatin Matrixes: A New Class of Hydrogels. Biomacromolecules.

[137] Bobula T., Buffa R., Procházková P., Vágnerová H., Moravcová V., Šuláková R., Židek O., Velebný V. (2016). One-pot synthesis of α,β-unsaturated polyaldehyde of chondroitin sulfate. Carbohydr. Polym..

[138] Panagos C., Thomson D., Bavington C.D., Uhrín D. (2012). Structural characterisation of oligosaccharides obtained by Fenton-type radical depolymerisation of dermatan sulfate. Carbohydr. Polym..

[139] Pierre G., Punta C., Delattre C., Melone L., Dubessay P., Fiorati A., Pastori N., Galante Y.M., Michaud P. (2017). TEMPO-mediated oxidation of polysaccharides: An ongoing story. Carbohydr. Polym..

[140] de Nooy A.E.J., Besemer A.C., van Bekkum H. (1995). Highly selective nitroxyl radical-mediated oxidation of primary alcohol groups in water-soluble glucans. Carbohydr. Res..

[141] Bragd P.L., Besemer A.C., van Bekkum H. (2000). Bromide-free TEMPO-mediated oxidation of primary alcohol groups in starch and methyl a-*D*-glucopyranoside. Carbohydr. Res..

[142] Jaušovec D., Vogrinčič R., Kokol V. (2015). Introduction of aldehyde vs. carboxylic groups to cellulose nanofibers using laccase/TEMPO mediated oxidation. Carbohydr. Polym..

[143] Parikka K., Nikkila I., Pitkanen L., Ghafar A., Sontag-Strohm T., Tenkanen M. (2017). Laccase/TEMPO oxidation in the production of mechanically strong arabinoxylan and glucomannan aerogels. Carbohydr. Polym..

[144] Moseley R., Waddington R., Evans P., Halliwell B., and Embrey G. (1995). The chemical modification of glycosaminoglycan structure by oxygen-derived species in vitro. Biochem. Biophys. Acta.

[145] Brown K.J., Hendry I.A., Parrish C.R. (1995). Evidence that carboxyl-reduced heparin fails to potentiate acidic fibroblast growth factor activity due to an inability to interact with cell surface heparin receptors. Exp. Cell Res..

[146] Gildersleeve J.C., Oyelaran O., Simpson J.T., Allred B. (2008). Improved Procedure for Direct Coupling of Carbohydrates to Proteins via Reductive Amination. Bioconj. Chem..

[147] Simi C.K., Abraham T.E. (2010). Physico chemical properties of aminated tamarind xyloglucan. Colloids Surf. B Biointerfaces.

[148] Koshida S., Suda Y., Arano A., Sobel M., Kusumoto S. (2001). An efficient method for the assembly of sulfated oligosaccharides using reductive amination. Tetrahedron Lett..

[149] Esposito E., Vlodavsky I., Barash U., Roscilli G., Milazzo F.M., Giannini G., Naggi A. (2020). Novel *N*-acetyl-Glycol-split heparin biotin-conjugates endowed with anti-heparanase activity. Eur. J. Med. Chem..

[150] Guerry A., Bernard J., Samain E., Fleury E., Cottaz S., Halila S. (2013). Aniline-catalyzed reductive amination as a powerful method for the preparation of reducing end-“clickable” chitooligosaccharides. Bioconj. Chem..

[151] Gulberti S., Lattard V., Fondeur M., Jacquinet J.C., Mulliert G., Netter P., Magdalou J., Ouzzine M., Fournel-Gigleux S. (2005). Phosphorylation and sulfation of oligosaccharide substrates critically influence the activity of human β1,4-galactosyltransferase 7 (GalT-I) and β1,3-glucuronosyltransferase I (GlcAT-I) involved in the biosynthesis of the glycosaminoglycan-protein linkage region of proteoglycans. J. Biol. Chem..

[152] Oshima T., Taguchi S., Ohe K., Baba Y. (2011). Phosphorylated bacterial cellulose for adsorption of proteins. Carbohydr. Polym..

[153] Feng H., Fan J., Yang S., Zhao X., Yi X. (2017). Antiviral activity of phosphorylated Radix Cyathulae officinalis polysaccharide against Canine Parvovirus in vitro. Int. J. Biol. Macromol..

[154] Song Y., Ni Y., Hu X., Li Q. (2015). Effect of phosphorylation on antioxidant activities of pumpkin (Cucurbita pepo, Lady godiva) polysaccharide. Int. J. Biol. Macromol..

[155] Wang X., Zhang Z., Yao Q., Zhao M., Qi H. (2013). Phosphorylation of low-molecular-weight polysaccharide from Enteromorpha linza with antioxidant activity. Carbohydr. Polym..

[156] Wu D., Wang Y., Li Y., Wei Q., Hu L., Yan T., Feng R., Yan L., Du B. (2019). Phosphorylated chitosan/CoFe_2_O_4_ composite for the efficient removal of Pb(II) and Cd(II) from aqueous solution: Adsorption performance and mechanism studies. J. Mol. Liq..

[157] Nishi N., Maekita Y., Nishimura S., Hasegawa O., Tokura S. (1987). Highly phosphorylated derivatives of chitin and chitosan as new functional polymers: Metal binding property of the insolubilized materials. Int. J. Biol. Macromol..

[158] Jayakumar R., Selvamurugan N., Nair S.V., Tokura S., Tamura H. (2008). Preparative methods of phosphorylated chitin and chitosan—An overview. Int. J. Biol. Macromol..

[159] Dadhich P., Das B., Dhara S. (2015). Microwave assisted rapid synthesis of N-methylene phosphonic chitosan via Mannich-type reaction. Carbohydr. Polym..

[160] Hermanson G.T. (2008). Bioconjugate Techniques.

[161] Bergman K., Elvingson C., Hilborn J., Svensk G., Bowden T. (2007). Hyaluronic Acid Derivatives Prepared in Aqueous Media by Triazine-Activated Amidation. Biomacromolecules.

[162] D’Este M., Eglin D., Alini M. (2014). A systematic analysis of DMTMM vs EDC/NHS for ligation of amines to hyaluronan in water. Carbohydr. Polym..

[163] Yang Y., Zhao Y., Lan J., Kang Y., Zhang T., Ding Y., Zhang X., Lu L. (2018). Reduction-sensitive CD44 receptor-targeted hyaluronic acid derivative micelles for doxorubicin delivery. Int. J. Nanomed..

[164] Freudenberg U., Sommer J.U., Levental K.R., Welzel P.B., Zieris A., Chwalek K., Schneider K., Prokoph S., Prewitz M., Dockhorn R. (2012). Using Mean Field Theory to Guide Biofunctional Materials Design. Adv. Funct. Mater..

[165] Freudenberg U., Hermann A., Welzel P.B., Stirl K., Schwarz S.C., Grimmer M., Zieris A., Panyanuwat W., Zschoche S., Meinhold D. (2009). A star-PEG-heparin hydrogel platform to aid cell replacement therapies for neurodegenerative diseases. Biomaterials.

[166] Palazon F., Benavides C.M., Leonard D., Souteyrand E., Chevolot Y., Cloarec J.P. (2014). Carbodiimide/NHS derivatization of COOH-terminated SAMs: Activation or byproduct formation?. Langmuir.

[167] Akaji K., Barker G., Bonewald L.F. (2004). Supplement. Houben-Weyl Methods of Organic Chemistry.

[168] Toida T. (2006). Method for Producing Alkyl-Esterified Glycosaminoglycan. United States Patent.

[169] Hirano K., Sakai S., Ishikawa T., Avci F.Y., Linhardt R.J., Toida T. (2005). Preparation of the methyl ester of hyaluronan and its enzymatic degradation. Carbohydr. Res..

[170] Šimkovic I., Mendichi R., Kelnar I., Filip J., Hricovini M. (2015). Cationization of heparin for film applications. Carbohydr. Polym..

[171] Siahaan P., Mentari N.C., Wiedyanto U.O., Hudiyanti D., Hildayani S.Z., Laksitorini M.D. (2017). The Optimum Conditions of Carboxymethyl Chitosan Synthesis on Drug Delivery Application and Its Release of Kinetics Study. Indones. J. Chem..

[172] Prestwich G., Zhang J., Kennedy T.P., Rao N. (2017). Use of Alkylated Semi-Synthetic Glycosaminoglycosan Ethers for the Treatment of Inflammation. United States Patent.

[173] Callegaro L., Renier D. (2009). Sulphated Hyaluronic Acid for Treating Degenerative Osteoarthritis. United States Patent.

[174] Venbrocks R., Roth A., Mueller P.-J., Moeller S., Ozegowski J., Peschel G. (2007). Use of Hyaluronic Acid Derivatives for Inhibiting Inflammatory Arthritis. US Patent.

[175] Dell A., Khoo K.-H., Panico M., McDowell R.A., Etienne A.T., Reason A.J., Morris H.R., Fukuda M., Kobata A. (1993). FAB-MS and ES-MS of Glycoproteins. Glycobiology: A Practical Approach.

[176] Huang R., Pomin V.H., Sharp J.S. (2011). LC-MS(n) analysis of isomeric chondroitin sulfate oligosaccharides using a chemical derivatization strategy. J. Amermican Soc. Mass Spectrom..

[177] Hu Y., Borges C.R. (2017). A spin column-free approach to sodium hydroxide-based glycan permethylation. Analyst.

[178] Ciucanu I., Kerek F. (1984). A Simple and Rapid Permathylation Method for the Permethylation of Carbohydrates. Carbohydr. Res..

[179] Sims I.M., Carnachan S.M., Bell T.J., Hinkley S.F.R. (2018). Methylation analysis of polysaccharides: Technical advice. Carbohydr. Polym..

[180] Cushnie T.P., Lamb A.J. (2011). Recent advances in understanding the antibacterial properties of flavonoids. Int. J. Antimicrob. Agents.

[181] Sales M.S., Roy A., Antony L., Banu S.K., Jeyaraman S., Manikkam R. (2018). Octyl gallate and gallic acid isolated from Terminalia bellarica regulates normal cell cycle in human breast cancer cell lines. Biomed. Pharmacother..

[182] de Cordova C.A., Locatelli C., Assuncao L.S., Mattei B., Mascarello A., Winter E., Nunes R.J., Yunes R.A., Creczynski-Pasa T.B. (2011). Octyl and dodecyl gallates induce oxidative stress and apoptosis in a melanoma cell line. Toxicol. Vitr..

[183] Ren J., Li Q., Dong F., Feng Y., Guo Z. (2013). Phenolic antioxidants-functionalized quaternized chitosan: Synthesis and antioxidant properties. Int. J. Biol. Macromol..

[184] Spizzirri U.G., Parisi O.I., Iemma F., Cirillo G., Puoci F., Curcio M., Picci N. (2010). Antioxidant–polysaccharide conjugates for food application by eco-friendly grafting procedure. Carbohydr. Polym..

[185] Ballell L., Joosten J.A.F., Maate F.A., Liskamp R.M.J., Pieters R.J. (2004). Microwave-assisted, tin-mediated, regioselective 3-*O*-alkylation of galactosides. Tetrahedron Lett..

[186] Hamer G.K., Perlin A.S. (1976). A 13C-N.M.R. Spectral Study of Chondroitin Sulfates A, B, and C: Evidence of Heterogeneity. Carbohydr. Res..

[187] Huckerby T.N., Lauder R.M., Brown G.M., Nieduszynski I.A., Anderson K., Boocock J., Sandall P.L., Weeks S.D. (2001). Characterization of oligosaccharides from the chondroitin sulfates. Eur. J. Biochem..

[188] Huckerby T.N., Brown G.M., Nieduszynski I.A. (1998). 13C-NMR spectroscopy of keratan sulphates. Eur. J. Biochem..

[189] Mulloy B., Forster M.J., Jones C., Davies D.B. (1993). N.m.r. and molecular-modelling studies of the solution conformation of heparin. Biochem. J..

